# What are the Challenges and Resilience Resources Identified by Informal Carers During the First UK COVID-19 Lockdown? A Longitudinal Qualitative Study Using Naturalistic Data

**DOI:** 10.1177/10497323221150131

**Published:** 2023-01-26

**Authors:** Warren J. Donnellan, Lily Sepulveda Garcia, Sarah M. Gibson, Paige Butcher, Matthew J. Lariviere

**Affiliations:** 1 Department of Psychology, 4591 University of Liverpool, Liverpool, UK; 2 Information School, 7315 University of Sheffield, Sheffield, UK; 3 School of Education, 7315 University of Sheffield, Sheffield, UK; 4 Brain Injury Rehabilitation Trust, 36012 Disabilities Trust, Liverpool, UK; 5 Centre for Research on Health and Social Care, School for Policy Studies, 1980 University of Bristol, Bristol, UK

**Keywords:** caring, COVID-19, challenges, resilience, longitudinal, qualitative

## Abstract

COVID-19 has posed serious challenges for informal carers living in the UK. This article examines some of the specific challenges facing carers and the resources they used to manage them throughout the first UK lockdown. We used a framework approach to analyse naturalistic, longitudinal data from 30 carers taking part in 96 of Mobilise’s daily Virtual Cuppas between March and July 2020. We found that lack of information and social restrictions cumulatively impacted carers’ sense of certainty, control and motivation. This took an emotional toll on the carers, leading to exhaustion and burden. However, carers quickly established new routines and used humour and self-care to actively manage their wellbeing. Carers received support but also provided it to those in need, including fellow members of the caregiving community, supporting an ecological approach to carer resilience. Our findings may be used to anticipate challenges and promote protective resilience resources in future lockdowns.

## Introduction

COVID-19 has posed serious challenges for informal carers living in the UK. Informal carers, defined as anyone who cares, unpaid, for a friend or family member who due to illness, disability, a mental health problem or an addiction cannot cope without their support (Carers Trust, 2021), already had an increased risk of stress and depressive symptoms (Joling et al., 2010) and lower levels of subjective wellbeing (Pinquart & Sörensen, 2003) and physical health (Carers UK, 2015; Vitaliano et al., 2003). However, data from the first lockdown in March–July 2020 revealed that not only did the total number of informal carers living in the UK increase from 9.1 million to 13.6 million (Carers UK, 2020a), but one-third of existing carers were now providing more care than before (Office for National Statistics, 2020). This increased demand left a significant proportion of carers feeling exhausted, unable to take breaks, and isolated from social support networks (Carers UK, 2020b).

The UK government sanctioned several lockdowns and restrictions between March 2020 and December 2021 (Institute for Government, 2022). Carer stress levels were highest during the first COVID-19 lockdown (Sheth et al., 2021). Park (2020) found that carers’ mental and physical health during this time varied based on caregiving status. Unsurprisingly, carers had poorer mental health and higher levels of fatigue than non-carers and long-term carers were more likely to experience a range of physical symptoms. This may be explained by the greater number of care hours and higher levels of care complexity reported by this group, which have both been associated with poorer health (Park, 2020; Tordorovic et al., 2020). Despite this, Chan et al. (2020) indicated that informal carers were largely overlooked by government policy during this time, forcing many to go without support, further exacerbating their health.

The closure of face-to-face support services played an important role in driving increased caregiving demand and subsequent negative health impacts. Orellana et al. (2020) note that services such as day care centres are a vital source of support and respite to care recipients and carers alike. For example, Willner et al. (2020) found that the closure of respite care services and schools for children and adults with intellectual disabilities led to increased burden, depression and anxiety in their carers. A growing body of research has been conducted investigating the impacts of COVID-19 on people living with dementia and their carers. Longitudinal research has revealed that social support usage dropped after the first UK lockdown was announced, resulting in increased depression in carers of people living with dementia (Giebel et al., 2021). Giebel et al. (2020a) found that domiciliary care services were able to continue running during lockdown, but many carers decided to discontinue this support due to perceived risk of COVID-19 infection, thus having to cover lost care hours themselves. In another study, Giebel et al. (2020b) identified three psychosocial impacts resulting from a perceived reduction in social support by unpaid dementia carers: loss of control; uncertainty and adapting to the ‘new normal’.

To date, COVID-19 social care research has taken a deficit approach and shown an almost universally negative impact on carers. However, there is robust population-based evidence to suggest that, although the incidence of mental health symptoms increased slightly during the early stages of the pandemic (March–April 2020), they decreased to pre-pandemic levels by mid-2020 (Robinson et al., 2022). In a study of people living with dementia and their carers during COVID-19, Hanna et al. (2022) found that people reported protective factors of resilience, including communication, adaptations, support networks, and lifestyle factors and coping mechanisms. These findings suggest that each person’s capacity to respond to and mitigate the challenges posed by COVID-19 is individualised and dependent on their protective resources. Whilst it is important to understand the risk factors and challenges facing carers, it is also important to identify factors that protect carers against stress.

**Figure 1.**
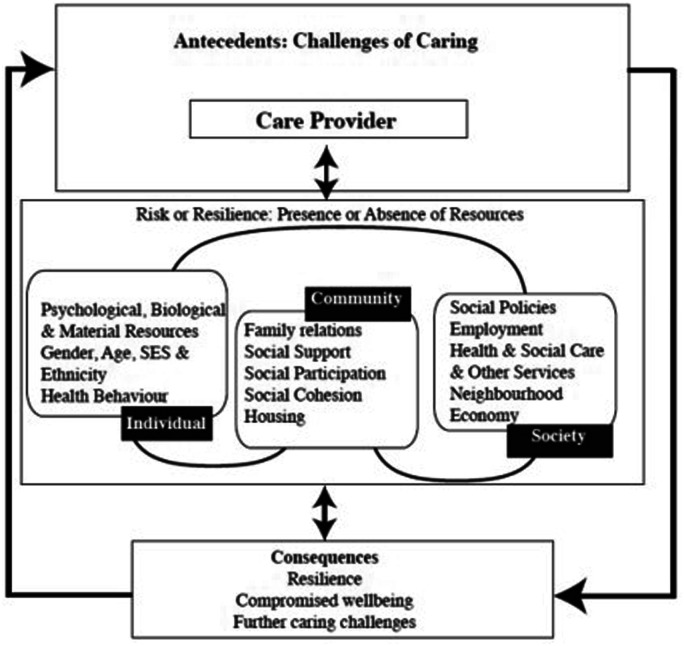
Ecological resilience framework applied to informal carers (Windle & Bennett, 2011).

Windle (2011) conducted a comprehensive concept analysis and defined resilience as: ‘The process of effectively negotiating, adapting to, or managing significant sources of stress or trauma. Assets and resources within the individual, their life and environment facilitate this capacity for adaptation and “bouncing back” in the face of adversity. Across the life course, the experience of resilience will vary’ (p. 163). Building on the notion that people draw on resources to facilitate their resilience, Windle and Bennett (2011) developed the ecological resilience framework (see Figure 1). The framework posits that carers draw on individual assets and community and societal resources which interact to help them manage the challenges of caregiving. Importantly, the resources are interactive, non-discrete and non-hierarchical; carers may draw on none, some or all of the resources at any one time, and the absence of resources may lead to further caring challenges or compromised wellbeing. Unlike other approaches, the ecological resilience framework emphasises systemic protective factors in addition to personal characteristics. As such, the burden of responsibility is not solely placed on the carer but also on community and societal systems, which has implications for carer support services and government policy. Although this model has been applied to informal carers of people living with dementia (Donnellan et al., 2015, 2017, 2019), it is less clear how it applies to informal carers more generally in the context of a global pandemic. In the current study, we adopt an ecological resilience approach to identify the resources that carers were drawing on to manage challenges during the first UK COVID-19 lockdown.

Researchers have identified numerous carer resilience resources (see Cherry et al., 2013; Teahan et al., 2018; Zhou, et al., 2020 for reviews within the context of dementia care). One area that has not been explored within the carer resilience literature, and yet has the potential to increase resilience in the context of social distancing, shielding and face-to-face support service closures, is technology-based support (Newman et al., 2019). Research has revealed that telehealth services during COVID-19, such as medical video conferencing and psychoeducation, were associated with improved resilience and wellbeing in both patients and carers alike (McGee et al., 2020; Su et al., 2020). One key mechanism through which technology-based services promote such positive outcomes is peer support. A scoping review of digital support for informal carers by Newman et al. (2019) showed that peer support increased awareness and understanding and brought carers together, promoting a sense of community, social inclusion and belonging. In a previous study of spousal dementia care, we found that the process of acquiring knowledge and sharing it with others ‘in the same boat’ contributed to resilience (Donnellan et al., 2015). When researchers and support providers work together to explore carers’ experiences they are better placed to deliver the most appropriate and effective evidence-based support now and in the future.

The COVID-19 pandemic and policy-related restrictions created new challenges for carers and disrupted their caregiving responsibilities. We know from the ecological resilience framework (Windle & Bennett, 2011) and our previous research with informal carers that there are both challenges and resources associated with caregiving (Donnellan et al., 2015, 2017, 2019). Using an ecological resilience perspective, this study uses longitudinal qualitative methods to analyse naturalistic secondary data to investigate the specific challenges of carers and the resources they used to manage them during the first UK lockdown. By using naturalistic secondary data, we gained an authentic, real-time insight into carers’ experiences that we could not have gained using other methods of primary data collection (Donnellan & Warren, 2022). We aimed to address the following two research questions: 1. What specific challenges were carers facing throughout the first UK lockdown in response to COVID-19? and 2. What resources were carers drawing on to manage these challenges?

## Methods

### Context

Mobilise describes itself as ‘the tech start-up by carers, for carers’. It is a community-driven business working with local authorities and carers’ centres to identify, engage and support carers at scale with innovative technologies. As the first UK lockdown commenced, Mobilise drew on existing research and established a series of daily ‘Virtual Cuppas’ (VC). Each VC is facilitated by a professional Carer Coach, and offers a relaxed, online setting for carers to connect with other carers around the country to discuss the day-to-day challenges they face. Mobilise adopts the following core principles in its VCs: identifying a topic or question in advance of each VC to structure the initial conversation; application of coaching principles to peer support discussion; guidance for participants on sustainable approaches to peer support; use of humour as a way to unlock difficult conversations and an ‘uplift’ activity at the end of each VC.

We are an interdisciplinary research team with expertise in social care research and qualitative methodology. The lead authors (WD and ML) were first approached by Mobilise in 2019 after they became aware of our published research on resilience and dementia care (WD) and digital support for older adults and their carers (ML). Whilst the research team did not have first-hand experience of caregiving themselves, it quickly became clear that we all shared a passion for supporting unpaid carers. As such, we formed a partnership and collaboratively decided on the research questions for this study. As ‘by carers, for carers’ was an underpinning ethos of Mobilise, we agreed that a qualitative methodology would be a good fit for the study, giving voice to the carers so that we could inform future research, policy and practice.

Mobilise’s VCs provided a large corpus of naturalistic secondary and longitudinal data that provide real-time insight into the challenges carers faced and the resources they used to manage those challenges. By following the same cohort of carers through the first national lockdown period, we gained a more dynamic picture of the challenges carers experienced. The longitudinal character of the data also helped us to identify changes in how carers accessed resources, including participation in the VCs themselves, and how they developed during the lockdown and initial easing of related restrictions.

### Methodology

As an interdisciplinary team of social scientists, the study design was informed by a social constructionist paradigm to qualitative inquiry where social reality is not located ‘out there’ but becomes ‘real’ through people’s individual and collective sense-making (Mason, 2002). To understand this process from the perspectives of the carers themselves, we adopted an interpretive approach to data analysis centred on how people understand themselves and their own situated experiences (Geertz, 2000). Distinctive from historical approaches to interpretive analysis from socio-cultural anthropology of situated immersion within a geographically bounded ‘culture’, our study relied on immersion within already textualised (Ricoeur, 1971) accounts of carers’ talk through their participation in VCs. Therefore, this study’s interpretive analysis was a form of secondary qualitative data analysis based on data collected longitudinally and naturalistically by Mobilise, the study’s industrial partner and data controller. To aid our analysis, we adopted a deductive approach to our interpretive analysis informed by the ecological resilience framework (Windle & Bennett, 2011). Our study uniquely draws on an innovative industrial partnership which enabled access to a granular and longitudinal dataset to explore challenges and resilience of carers during the first national lockdown period in the UK.

### Data

Qualitative longitudinal, naturalistic data were drawn from 96 of Mobilise’s daily VCs, between 20th March 2020 and 28th July 2020. Each VC lasted 30 min, providing approximately 49 h of data to analyse. As the data were not collected specifically for research purposes, we were able to gain a more naturalistic insight into the challenges and resources that carers faced over a 4-month period during the first national lockdown.

We used the consolidated criteria for reporting qualitative studies (COREQ) guidelines (Tong et al., 2007) as a starting point to guide our thinking about matters pertaining to rigor in relation to the study while keeping focus on the thinking behind the criteria - not simply ticking them off on a list.

Our study was approved in April 2020 by the University of Sheffield Faculty of Social Sciences Research Ethics Committee (approval number 034306). All VC participants provided informed consent to our industrial partner, Mobilise, for their data to be routinely collected and processed. The consent process involved VC participants completing a short online form created by Mobilise. Mobilise entered into a data sharing agreement with the first and last author’s institutions at the time of the study to analyse this secondary dataset. The research team (i.e., authors of this article) did not have contact with any participants of the VCs. Only data from VC participants who consented to its use were made available to the research team.

### Participants

A total of 30 participants took part in the VCs between 20^th^ March and 28^th^ July 2020, with a maximum attendance of 16 carers and minimum attendance of one carer in each VC. The average attendance per VC was approximately six carers. Demographic data were collected by way of an optional short survey published by Mobilise in September 2020. The survey revealed a mix of ages and ethnicities, with the majority of VC participants being white, aged 46–65 and living across England and Wales, including North West; South West; Midlands; South East; South Central and South Wales. Individual frequency data per region were not available. Most VC participants reported that they had been providing care to a loved one for more than 1 year and that care frequency had increased since the start of lockdown (see Table 1). Whilst it was not possible to align these demographic data to our findings without compromising the anonymity of VC participants, the data do bring some broad context to our findings.

**Table 1.** Demographic Characteristics of VC Participants (*N* = 30).

Participant characteristics	*n*	% of *N* = 30
Age		
18–45	4	13.3
46–65	21	70
66+	5	16.6
Ethnicity		
Asian	2	6.7
Black	2	6.7
White	25	83.3
Prefer not to say	1	3.3
Care duration		
5–11 months	2	6.7
1+ years	27	90
Reduced/stopped caregiving due to COVID-19	1	3.3
Care frequency		
Reduced since lockdown	1	3.7
Same since lockdown	4	14.8
More since lockdown	22	81.4

### Method of Analysis

We analysed VC data using a longitudinal framework analysis (Ritchie et al., 1994). We adopted a framework approach because we know from the ecological resilience framework (Windle & Bennett, 2011, see Figure 1) and our subsequent research with informal carers that there are both challenges and resources associated with caregiving (Donnellan et al., 2015, 2017, 2019). Using the ecological resilience framework (Windle & Bennett, 2011), we coded for the specific challenges and resources faced by carers throughout the first UK COVID-19 lockdown. The longitudinal nature of this approach allowed us to gain a deeper understanding of carers’ experiences including how and why they changed over time.

The analysis was primarily conducted by three female Research Assistants (RAs), PB, LS and SG, and supervised by two male qualitative researchers, ML and WD, for independent coding purposes. All members of the research team were experienced in the use of qualitative research methodologies. The coding framework was managed using a Microsoft Excel spreadsheet comprising the coding for each analysis, alongside VC number, VC date and the topical question posed by the Facilitator during that session. Importantly, the topical questions did not ask specifically about challenges and resources. Instead, topical questions included: ‘what is your superpower?’; and ‘what is the biggest difference since lockdown started?’ We included socio-political contextual information alongside each VC, including key events and government announcements regarding COVID-19. Rather than explicitly drawing out socio-political context in our findings, we used it as a form of triangulation in our analysis to facilitate deeper understanding of the data and ensure that the conclusions drawn were ultimately more rigorous. Each VC was hyperlinked to the corresponding transcript in Microsoft Word, where VC participant quotations were tabulated alongside exploratory notes and codes. These methods ensured that the process of analysis was fully transparent.

In line with Holland et al. (2006), we conducted our qualitative longitudinal analysis in two parts: an initial cross-sectional ‘within-time’ analysis and a longitudinal ‘between-time’ analysis:

#### Within-Time Analysis

The RAs first read the transcripts several times to ensure familiarisation with the data. The transcripts were then evenly distributed between PB, LS and SG. The full research team met regularly to develop a provisional list of codes and ensure the constant comparison of new data. Over the course of analysis, the RAs added any new codes and removed any codes that became superfluous. In order to further strengthen the rigour of the findings, a subset of VC transcripts were independently re-coded by the other RA. Any coding discrepancies were discussed until a consensus was reached. The full research team agreed when data saturation had occurred; this was when all researchers agreed that no further patterns or themes were being observed in the data (O’Reilly & Parker, 2013). Codes were then reviewed and consolidated into broader themes relating to the key challenges that carers were facing and the resources they were drawing on to manage these challenges. The final themes were presented to the wider research team who discussed the extent to which they were grounded in the data and identified any omissions or ambiguities in the analysis.

#### Between-Time Analysis

Once themes had been generated from individual VCs, the RAs conducted a secondary between-time thematic analysis of all VC themes. This allowed us to identify the dynamic trajectory of challenges and resources over the course of the first COVID-19 lockdown period. We followed the same systematic processes outlined in the section above.

## Findings

**Figure 2.**
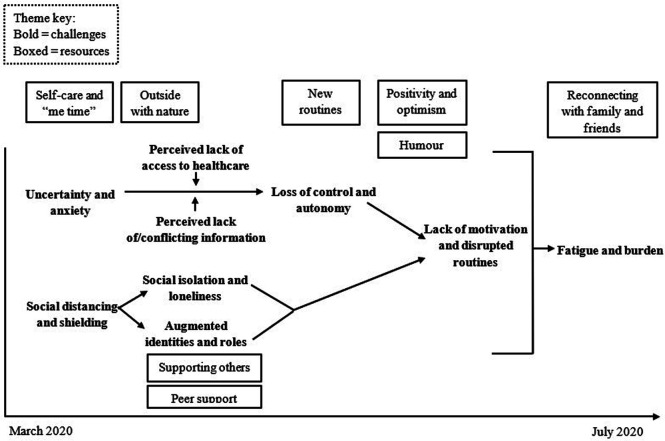
Linkages within and between analytical themes over time. *Note*. Positioning of themes on the timeline is not intended to be exact and is for illustrative purposes only.

The findings below are presented in two sections containing themes linked to each of the study’s research questions. Due to the constraints of the data source, we were not able to identify individual participants across all VCs. Instead, we present illustrative quotations alongside the date of the corresponding VC. See Figure 2 for a summary of key analytical themes over time.

### What Specific Challenges were Carers Facing throughout the First UK Lockdown in Response to COVID-19?

The announcement of a national lockdown on 23rd March 2020 had a profound negative impact on the carers taking part in the VCs. In the early stages of lockdown, the carers experienced a great deal of uncertainty and anxiety in terms of what the lockdown would mean for them and their loved ones’ functioning. This is illustrated in the following quote, just 2 days after the lockdown was announced:

“My biggest fear, because she has mixed dementia, is that by the time it is all lifted, my Mum will have forgotten me” (VC 25/03/2020).

As time passed, the carers’ uncertainty led to a perceived loss of control and autonomy and increased apprehension about what the future held. The following carer used an interesting analogy to capture her experience on 23rd April 2020:

“I feel as if I’m on a wave, you know, and you do not know where that wave is going to throw you next… I’m sort of bracing myself to see where we go next” (VC 23/04/2020)

These feelings of uncertainty and loss of control were exacerbated by perceived ambiguity and lack of information from the national government. Two months into the lockdown, the following carer highlighted her specific concerns over returning to work:

“We go back to normal, what’s that gonna look like? My job might be open, by my support networks might not be, if that makes sense… I sort of go, are we fully back? Are we not back? What if I can’t go back straight away? (08/05/2020).

Around the same time, other carers reported feeling confused by conflicting information from different sources, for example:

“They’ve told me to isolate from my house as well. But no one sent me a medical, it’s only what the nurse had been telling me. I don’t know what to do best for my husband or myself. So um, I’m fairly confused” (12/05/2020).

A lot of the anxiety caused by uncertainty and lack of information centred on perceived access to essential healthcare services. The following quote from May 2020 highlights the carer’s concerns:

“They’ve got nothing over here. There’s no ventilators, no oxygen, you know… they’ve got like nothing” (15/05/2020)

In addition to feelings of uncertainty, the carers felt restricted by government-sanctioned measures such as social distancing and shielding. In the early stages of lockdown, many carers reported social isolation and feelings of loneliness. The following carer captured her experience of restriction and isolation using another analogy:

“It’s like a prison. The walls are closing in” (26/03/2020).

As time passed, isolation and loneliness led to a desire for social interaction. The following quote from July 2020 illustrates the carer’s desire for affectionate contact and proximity from loved ones that they still could not see:

“I’ve been on my own and locked down as well. A lot of us have, so, you know, I just said one day ‘it’d be nice for someone to make me a cup of tea’” (06/07/2020).

The cumulative impact of restriction and perceived lack of information led to carers feeling less motivated than usual. The following carer took a negative view of her reduced motivation:

“I’ve been rubbish the last couple of days. Not wanted to do very much. I’ve slept a lot, which is not like me” (30/04/2020).

As time passed, the carers’ feelings of demotivation challenged existing daily routines that were important to the smooth running of their household. The following quote from June 2020 illustrates the impact of demotivation on routine:

“I’ve struggled today. I’ve only just got around to making my bed and I haven’t made it properly. I’ve not been very mobile today at all. I have to come to terms with the fact that it is going to be like that for a while” (03/06/2020).

For many carers, the augmented identities and roles of carer and wife/husband, or carer and daughter/son, had always been challenging to negotiate. However, the national lockdown and increased time spent at home posed additional challenges to the carers. In the following quote from May 2020, the carer describes how the line blurred between work, home and care:

“There’s no kind of physical divide… and it’s kind of all merging into one… So, for me, it’s about managing that work from home and making sure that I’ve got time for everything, including that work-life balance” (15/05/2020).

All of these challenges took an emotional toll on the carers taking part in the VCs. By June and July 2020, there was a clear cumulative impact of 3 to 4 months’ worth of restrictions and increased caregiving responsibilities. This led to feelings of fatigue in the carers and a perceived inability to fulfil all of their responsibilities, for example:

“I couldn’t figure out whether I was ok and just tired… what would make it better for me is having more arms, more legs, more time and more of me really” (10/06/2020).

Increased fatigue was associated with increased feelings of burden and burnout in the carers, as the following carer described during one of the latter VCs in July 2020:

“Even though he’s not here in presence he still takes up an awful lot of my energy and my time… my brain is fried and I can’t think… I just need to have some me-time” (23/07/2020).

### What Resources were Carers Drawing on to Manage these Challenges throughout the First UK Lockdown?

Carers taking part in the Mobilise VCs quickly began to draw on their assets and resources to manage the challenges posed by the COVID-19 lockdown restrictions. For example, despite feeling demotivated in the early part of lockdown, the carers established a new set of routines. The following quote illustrates how establishing structure and routines helped the carer to regain a sense of control just days into the lockdown being announced in March 2020:

“Routine is key for us at the moment. We’ve got like a semi-structure if you like. But if something doesn’t happen, that’s ok. If we’ve got that structure, it keeps us sane” (26/03/2020).

Around the same time, carers started to use more active methods of dealing with the lockdown. For example, some carers began to accept the lockdown and viewed their situation more positively and optimistically:

“Although this is a very real situation. There is some hope and it’s not all doom and gloom” (25/03/2020).

The carers were not only able to stay positive, but many were able to use humour to deal with the challenges they were facing. This reflected the fact that the VCs were in a safe environment where the carers could be themselves. The ability to see the funny side of things was an essential resource for the carers throughout the whole lockdown period, as illustrated by the following quote from April 2020:

“How do I switch off? I tend to have a little word with my boyfriend and he makes me laugh and that helps me relax before I go to bed. So I like doing that. He’s my therapy” (28/04/2020).

The carers also began to manage their frustrations and started to make small changes to look after themselves. Self-care was an important part of this, as the following quote demonstrates:

“I decided on a whim yesterday while I was getting my essential shopping to buy some hair dye. So I’ve got a nice vibrant purple” (30/03/2020)

Some of the carers spent their ‘me time’ practicing relaxation techniques to manage the uncertainty and anxiety associated with lockdown. In the following quote from April 2020, the carer describes how they use principles of mindfulness and meditation to relax:

“I don’t have access to a garden, but I can see a garden outside my window. So I use it for like meditation and stuff and being mindful. So I watch the birds and the bees and the wind and the trees. That’s one thing that helps me relax” (02/04/2020).

Spending time outside with nature, whether that be in a home garden or on a government-sanctioned walk around the park, became a common way for carers to manage their stress levels, for example:

“I think Tuesday’s supposed to be beautiful. So I’m going to be doing things in my garden tomorrow. If the weather allows anyway” (04/04/2020).

In addition to their primary caregiving roles, carers received and provided support to others. In the following quote from April 2020, the carer highlighted the exchange of support to their neighbours and friends:

“Not a lot we can do about the coronavirus but if you can help your neighbour or them help you out, it makes you feel good” (01/04/2020).

Peer support in particular became one of the most crucial resources for the carers around this time. The lockdown made face-to-face support between carers impossible but that did not stop them from helping each other out online. The following carer described the nature of this peer support:

“We are on a community Facebook page full of ‘moaners’. Generally when somebody wants to say something, we say look, this person needs help” (31/03/2020).

This sense of community had a profound effect on the carers’ wellbeing and spurred them on to continue looking after their loved ones, as the following quote illustrates:

“I think that’s what’s keeping me going actually is just knowing that the community is pulling together out there and properly falling over themselves trying to do stuff… I feel that if I need something, there’s a multitude of people I can rely on” (21/03/2020).

As time went on and lockdown restrictions began to ease, the carers started to reconnect with family and friends. The following quote from June 2020 highlights the impact of reconnecting on the carers’ wellbeing:

“It was lovely to see my friends face-to-face as opposed to being on FaceTime or whatever… It was a good day for me, even though I’ve cried” (18/06/2020).

## Discussion

Our first research question asked: what specific challenges were carers facing throughout the first UK lockdown in response to COVID-19? We found that a perceived lack of information and social restrictions had a cumulative impact on carers’ sense of certainty, control and motivation. Over time, this took an emotional toll on the carers, leading to feelings of exhaustion and burden. These findings are in line with other research which found carers experienced increased stress (Sheth et al., 2021), increased fatigue (Park, 2020), and reduced social support as a result of the first COVID-19 lockdown (Carers UK, 2020b). The negative impacts seen in our study are not necessarily a result of new challenges; indeed, research shows that informal carers already experienced increased physical (Carers UK, 2015; Vitaliano et al., 2003) and mental health problems (Joling et al., 2010; Pinquart Sörensen, 2003) pre-lockdown. Rather, lockdown compounded existing caregiving challenges. This may reflect the higher proportion of VC participants providing relatively more care hours over an extended period, which we know is associated with poorer health (Park, 2020; Tordorovic et al., 2020).

Perceived lack of access to healthcare services was a significant challenge for VC participants. This is in line with Giebel et al. (2021), who found that carers’ participation in social support services reduced during the first UK lockdown. In addition to existing evidence that such service closures resulted in increased depression, anxiety and burden (Giebel et al., 2021; Willner et al., 2020), we found that it also led to isolation and loneliness. Unsurprisingly, the situation was exacerbated by the carers’ perception of ambiguity from government relating to when essential services would reopen (Chan et al., 2020). Indeed, the uncertainty and loss of control reported by VC participants may be a direct impact of reduced social support rather than the lockdown itself (Giebel et al., 2020b).

Our second research question asked: what resources were carers drawing on to manage challenges throughout the first UK lockdown? Despite being physically restricted, we found that carers established new routines and adopted positive strategies such as humour and self-care to actively manage their wellbeing. Carers both received and provided support to those in need; not just their loved ones, but also members of the caregiving community. A lot of existing research on carer resilience has focused on dementia care (Cherry et al., 2013; Donnellan et al., 2015, 2017, 2019; Teahan et al., 2018; Zhou et al., 2020). Our findings demonstrate that the response of carers to the first COVID-19 lockdown was heterogeneous; contrary to the focus of much research, it was not a universally negative experience. In fact, there was evidence of resilience and protective resources being used to manage the challenges associated with COVID-19. This is in line with recent quantitative (Robinson et al., 2022) and qualitative research (Giebel et al., 2020b) emphasising resilience and adaptation in the face of COVID-19. In line with previous research, peer support was a key resource for carers taking part in the VCs. Both the provision and receipt of support with fellow carers was highly valued and created a sense of community which protected against the challenges posed by the COVID-19 lockdown (Donnellan et al., 2015; Newman et al., 2019).

Using a social constructivist paradigm, we adopted a deductive approach to our interpretive analysis informed by the ecological resilience framework (Windle & Bennett, 2011). Whilst this approach meant that we could not generate a framework inductively and precludes us from discussing our findings beyond existing theory, it did enable us to test and potentially extend the resilience framework in a different context to previous research. We confirmed existing knowledge by identifying resources that we know are associated with carer resilience in a pre-pandemic context; that is, positivity, optimism, supporting others, peer support and routine (Donnellan et al., 2015, 2017, 2019). However, we also extended the resilience framework by identifying novel resources that are unique to the COVID-19 context; that is, self-care/‘me time’, being outside with nature, reconnecting with family and friends. These resources emerged at individual (e.g., positivity and optimism), community and societal levels (e.g., reconnecting with family and friends) which suggests that carer resilience transcends the individual and involves a wide range of protective factors. These protective factors are important as research shows that they can promote positive outcomes and protect against negative ones (Jones et al., 2019; Kalisch et al., 2015; van Breda, 2018). Finally, our longitudinal design added a novel temporal dimension to the ecological resilience framework as we got a sense of how resources evolved over time in response to changing care demands. For example, early in the first lockdown, self-care and ‘me time’ was important as there was a lot of uncertainty and anxiety. As time passed, the provision and receipt of support became important to combat isolation and feelings of loneliness. Towards the end of the first lockdown period, reconnection with family and friends balanced feelings of fatigue and burden. This suggests that resilience is a dynamic process; carers utilise different resources at different times for different challenges. There is no ‘one size fits all’ approach to resource use for carers.

The main contribution of this research is our innovative use of longitudinal, naturalistic secondary data provided by the technology-based carer support service, Mobilise. The naturalistic quality of the data increased the likelihood that carers’ experiences were genuinely captured and not a potential artefact of a survey or interview schedule. The longitudinal quality of the data also allowed us to explore how carers experienced changes over the duration of the first UK lockdown. Of course, the study was also limited by its exclusive analysis of naturalistic, secondary data. We did not have the opportunity to clarify any of the statements that VC participants made. The extent to which individual VC participants may have been influenced by the facilitator’s topical questions, statements from other group members, or the fact that the VCs were being recorded was also unknown. Although none of the VCs posed questions relating specifically to challenges or resources, it is possible that some of the topical questions may have primed the carers’ responses in some way, for example., ‘what is your superpower?’ could have created a sense of humour which is one of our themes. The study would therefore have benefitted from supplementary primary data collection, such as additional interviews with carers participating in the VCs, to triangulate the findings and thus increase the rigour of the research.

The findings presented here do not fully represent the experiences of carers during the first UK lockdown. The demographic characteristics of our sample demonstrate some diversity but are still relatively narrow. The demographic survey collected only a small amount of demographic information from the carers which meant there was a lot we did not know about the sample, for example, carers’ living arrangements in relation to their loved one(s). Because the survey was conducted after the VC period ended, we were also unable to link our findings to specific carers or demographic characteristics. Future research should undertake complementary prospective data collection to gather a more comprehensive set of demographic data to help contextualise findings. As indicated earlier, carers are heterogeneous in that not all face the same challenges nor experience the same uplifts. Indeed, there is increasing evidence of a ‘digital divide’ that disadvantages certain groups who lack the infrastructure and skills to effectively utilise digital technology in their everyday lives (Lariviere et al., 2020). Therefore, the current findings may only apply to the most digitally capable carers and may not be meaningfully transferred to less digitally capable carers. Future research should consider how technology-based support services such as Mobilise can most effectively support carers.

Unfortunately, COVID-19 remains with us. Since the first national lockdown ended, the UK continued to experience various levels of localised restrictions and two further national lockdowns (Baker et al., 2021). Informal carers will continue to face challenges to their wellbeing and capability to provide care both now and in the future. Whilst additional research is necessary to explore carers’ experiences throughout the whole pandemic, our findings have useful implications and applications. Using our findings, carer support services, local authorities and national government are able to anticipate the anxiety, social isolation and loneliness facing carers during lockdown and intervene early to prevent escalation to a loss of control, autonomy and motivation. The government should ensure that its messaging is targeted appropriately and is consistent and tailored to carers’ unique needs to avoid confusion, for example, which carer support services are still running during lockdown periods. Increased opportunities for respite should be provided to carers to reduce their fatigue, burden, and burnout as lockdowns progress, during which time carers should be encouraged to prioritise their own wellbeing and ‘me time’. Finally, peer support forums should be encouraged so that carers can support each other and share what works or does not work for them, e.g., adopting new routines, humour, spending time outside with nature.

In conclusion, our findings identified the challenges faced and resources used by carers participating in the Mobilise VCs during the first COVID-19 lockdown in the UK. Informal carers faced numerous challenges pre-COVID-19, and the first lockdown further compounded these challenges. However, the carers used individual, community and societal resources to manage these challenges. Whilst further prospective, primary research capturing the carers’ full demographic context is necessary to complement this work, the findings have important implications for policy and practice in this and any future pandemics.
